# 

**Published:** 1890-11-22

**Authors:** 


WITH EXTRA NURSING SUPPLEMENT.
Per Annum, 6s. 6d.; post free.
Monthly Parts, 6d.; post free 8d.
Ditto per Annum, 18s. post free.

### THE HOSPITAL

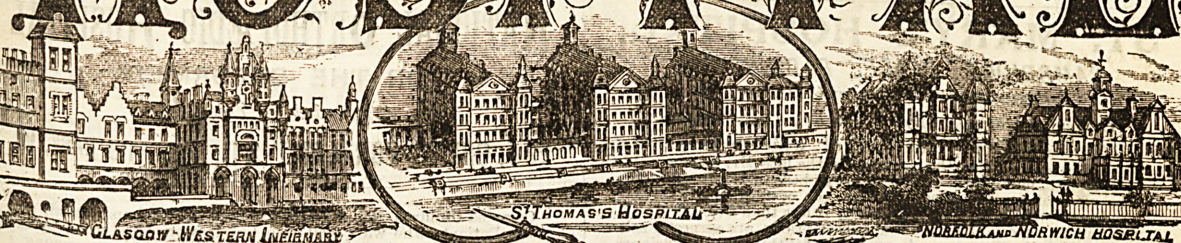

A WEEKLY JOURNAL OF

### Science, Medicine, Nursing and Philanthropy.

### SATURDAY, NOVEMBER 22nd, 1890.

**Koch's Cure: its Nature and its Limits** - 117

**Passing Topics.**—Pensions for Hospital Workers.—I. - 118

**Turning Over New Leaves.**—Mr. Besant on Drunkenness 119

**Working Women in Large Towns. XVI.**—Social, Moral, and Physical Aspects of the Subject - 119

**Everybody's Page** - 120

**The Doctor's Armchair.**—A New Champion of Medical Women's Rights - 121

**The History and Capabilities of Herbal Simples.** By W. T. Fernie, M.D. XXIII. Cowslip - 122

**Medical Syndicates** - 123

**Notes and News**, 124; **Scraps and Gleanings** - 125

**Annotations.**—"Charlotte Martin, Hospital Nurse"—Hospital Chaplains—Nurses and their Pay - 126

**The Practitioner's Mirror and Retrospect:**—

The Post-Graduate Course at the Brompton Hospital—Tubercular Affection of the Larynx - 127

The London Post-Graduate Course at the Paddington Hospital.—Pathology - 128

Dr. Koch on Tuberculosis. - 129

**The Student of Medicine.**—Editorial Notes—Appointments—Students' Medical Societies—The Student's Bookshelf—Letter to the Editor—Pass Lists—Athletics - 132

**General Charities.**

### EXTRA SUPPLEMENT—THE NURSING MIRROR.

**En Passant.**—Nurses' Inventions—The Special Pro.—Catholic Nurses—That Unhappy Country—The Sick Nurse

**Lectures on Surgical Ward Work and Nursing.** By Alex. Miles, M.B. (Edin.), C.M., F.R.C.S.E.—Lecture IV.—Antiseptics in Common Use (*continued*) - xxxvi

**Examination Questions**—Prize Answer for October - xxxvi

**The Nurses' Bookshelf.**—Asylum Attendants—A Royal Example—A Practical Handbook - xxxvii

**For Reading to the Sick.**—To Hospital Patients - xxxvii

**Nurses on Their Travels.**—The Golden City - xxxviii

**Everybody's Opinion.**—Six Months in a Hospital—Male Nurses—Post as Attendant—Prayer-books for Patients, xxxviii.; Asylum Attendants — Nurses' Watches - xxxix

**Appointment—Vacancies** - xxxix

**Notes and Queries**, xxxix; **Our Understandings** - xl

**Where to Go—What to Read** - xl

**Amusements and Relaxation** - xl

